# A Novel Exosome-Relevant Molecular Classification Uncovers Distinct Immune Escape Mechanisms and Genomic Alterations in Gastric Cancer

**DOI:** 10.3389/fphar.2022.884090

**Published:** 2022-06-03

**Authors:** Yubiao Lin, Kaida Huang, Zhezhen Cai, Yide Chen, Lihua Feng, Yingqin Gao, Wenhui Zheng, Xin Fan, Guoqin Qiu, Jianmin Zhuang, Shuitu Feng

**Affiliations:** ^1^ Department of Oncology, Xiamen Haicang Hospital, Xiamen, China; ^2^ Department of General Surgery, Xiamen Haicang Hospital, Xiamen, China; ^3^ Chenggong Hospital Affiliated to Xiamen University, Xiamen, China

**Keywords:** gastric cancer, exosomes, immunotherapy, tumor microenvironment, immune escape

## Abstract

**Objective:** Gastric cancer (GC) is a highly heterogeneous malignant carcinoma. This study aimed to conduct an exosome-based classification for assisting personalized therapy for GC.

**Methods:** Based on the expression profiling of prognostic exosome-related genes, GC patients in The Cancer Genome Atlas (TCGA) cohort were classified using the unsupervised consensus clustering approach, and the reproducibility of this classification was confirmed in the GSE84437 cohort. An exosome-based gene signature was developed *via* Least Absolute Shrinkage and Selection Operator (LASSO) regression analysis. Immunological features, responses to immune checkpoint inhibitors, and genetic alterations were evaluated *via* computational methods.

**Results:** Two exosome-relevant phenotypes (A and B) were clustered, and this classification was independent of immune subtypes and TCGA subtypes. Exosome-relevant phenotype B had a poorer prognosis and an inflamed tumor microenvironment (TME) relative to phenotype A. Patients with phenotype B presented higher responses to the anti-CTLA4 inhibitor. Moreover, phenotype B occurred at a higher frequency of genetic mutation than phenotype A. The exosome-based gene signature (GPX3, RGS2, MATN3, SLC7A2, and SNCG) could independently and accurately predict GC prognosis, which was linked to stromal activation and immunosuppression.

**Conclusion:** Our findings offer a conceptual frame to further comprehend the roles of exosomes in immune escape mechanisms and genomic alterations of GC. More work is required to evaluate the reference value of exosome-relevant phenotypes for designing immunotherapeutic regimens.

## Introduction

Gastric cancer (GC) is one of the most common malignant carcinomas diagnosed globally, seriously jeopardizing human health (Bray et al., 2018). Despite the remarkable progress of therapies, the 5-year overall survival (OS) of advanced patients is merely 20% (Chen Y. et al., 2021). Cisplatin, paclitaxel, 5-fluorouracil, and doxorubicin remain the main chemotherapeutic agents against GC. Nevertheless, chemotherapeutic resistance is a common cause of recurrence and metastases of GC (Lin et al., 2020). Immunotherapy represented by immune checkpoint inhibitors (ICIs) against PD-1/L1 and CTLA-4 presents the durable therapeutic effects for the minority of GC patients; undesirably, most patients cannot respond to it (Shitara et al., 2018; Shitara et al., 2020; Janjigian et al., 2021). Thus, a more effective systemic treatment is urgently required.

Exosomes, a subgroup of extracellular vesicles with 30–150 nm, are secreted by nearly all cell types, which transmit cellular molecular components [protein, DNA, lipid, messenger RNA (mRNA), and non-coding RNA, etc.], thereby promoting cell-to-cell communication (Tang et al., 2021). Tumor progression is regarded as a multistep process, and accumulated evidence has suggested that the tumor microenvironment (TME) in which tumor cells grow and survive also exerts an important role in tumor progression (Zeng et al., 2019). Tumor cells elicit diverse alterations in biological behaviors *via* directly or indirectly interacting with the TME components (Jiang et al., 2019). Exosomes mediate the communications between the TME and tumor cells. For instance, tumor-associated macrophage-released exosomes facilitate the migration of GC cells through transferring apolipoprotein E (Zheng et al., 2018). Exosomal miR-451 released by GC cells enhances T-helper 17 cell differentiation (Liu F. et al., 2018). Recurrence and metastases are the main obstacles to favorable survival outcomes of GC (Chen D. et al., 2021). Tumor cells break away from the primary cancer nest and enter the circulatory system *via* blood vessels or lymph vessels, thereby facilitating tumor metastases (Cai et al., 2020). For avoiding the blockage of the extracellular matrix (ECM) and promoting the remodeling of the tumor-friendly TME, tumor cells release biologically active factors to elicit the communications of tumor cells with stromal subsets, thereby creating a favorable condition for cancer metastases (Sathe et al., 2020). Exosomes exert a crucial role in this process *via* carrying DNA, lipid, or ncRNA (Zhang et al., 2021). For instance, lymph node metastasis-GC cells educate mesenchymal stem cells through exosomal Wnt5a-triggered activation of the YAP pathway (Wang et al., 2021). In addition, exosomes released by tumor cells can hinder the activation of the immune system and the development of immune cells, thereby blocking the immune defense mechanism of tumor cells and eliciting the immune escape mechanism. Moreover, experimental evidence has demonstrated that exosomes participate in the chemotherapeutic resistance of GC (Lin et al., 2020). For instance, exosomes carrying miR-500a-3p trigger cisplatin resistance and stemness through the negative modulation of FBXW7 in GC (Lin et al., 2020). Tumor-associated macrophage-derived exosomal CRNDE attributes to cisplatin resistance in GC (Xin et al., 2021). The molecular subtype classification of GC provides an opportunity for personalized therapy. Thus, it is of significance to comprehensively recognize the exosome-relevant molecular classification in GC. This study conducted two exosome-relevant phenotypes with distinct immune escape mechanisms and genomic alterations in GC.

## Materials and Methods

### Data Acquisition

This study collected three gene expression profile cohorts for GC: TCGA-STAD (*n* = 350) from TCGA (https://portal.gdc.cancer.gov/), GSE84437 (*n* = 433) (Yoon et al., 2020), and GSE15459 (*n* = 192) (Muratani et al., 2014) from the GEO repository (https://www.ncbi.nlm.nih.gov/gds/). RNA-seq data (FPKM value) of TCGA-STAD cohort were downloaded from the Genomic Data Commons (https://portal.gdc.cancer.gov/) using the TCGAbiolinks package (Colaprico et al., 2016). FPKM value was transformed to TPM value. For the GSE84437 cohort on the Illumina platform, the normalized matrix files were directly downloaded. For the GSE15459 on the Affymetrix platform, the raw “CEL” files were downloaded, which were normalized utilizing a robust multi-array averaging approach. For TCGA-STAD cohort, somatic mutation and copy number variation (CNV) profiles were also retrieved. Through reviewing the published literature, we collected 121 exosome-related genes, as listed in Supplementary Table S1.

### Unsupervised Consensus Clustering Analysis

The ConsensusClusterPlus package was applied for consistent clustering and determining exosome-relevant phenotypes on the basis of expression profiling of prognostic exosome-related genes derived from univariate Cox regression analysis (*p*-value < 0.05) (Wilkerson and Hayes, 2010). Through the Euclidean squared distance metric and the K-means clustering approach, GC specimens were classified as k clusters from k = 2 to 9. Approximately 80% of the specimens were chosen at each iteration. Following 100 iterations, the classification results were acquired, which were visualized into the heatmaps of the consensus matrix. The optimal number of clusters was identified in accordance with a cumulative distribution function (CDF) plot and an item tracking plot. The accuracy of this classification was verified through principal-component analysis (PCA). The classification was externally verified in the GSE84437 cohort.

### Gene Set Variation Analysis

The GSVA package was used for exploring the potential biological functions and progress variations of each phenotype (Hänzelmann et al., 2013). The Hallmark gene sets were derived from the Molecular Signatures Database (Liberzon et al., 2015).

### Evaluation of Immunological Status

The relative abundance of each immune cell component within the TME was quantified *via* applying the single-sample Gene Set Enrichment Analysis (ssGSEA) approach. The marker gene sets of 28 immune cell types were acquired from Bindea et al. (2013). The immunomodulators that comprised MHCs, receptors, chemokines, and immune-stimulators and immune-inhibitors were curated from the study of Charoentong et al. (2017). Moreover, known immune checkpoints were retrieved from Auslander et al. (2018). Mariathasan et al. established the gene sets of immune (CD8^+^ T effector, antigen processing machinery) and stromal [pan-fibroblast TGFb response signature (Pan-F-TBRS), epithelial-mesenchymal transition (EMT), and angiogenesis] pathways. Their levels were quantified with the ssGSEA.

### Evaluation of Responses to Immune Checkpoint Inhibitors

The Tumor Immune Dysfunction and Exclusion (TIDE) algorithm was applied for predicting the responses to ICIs (Jiang et al., 2018). This computational method was implemented on the basis of two tumor immune escape mechanisms: inducing T-cell dysfunction in tumors with increased infiltration of cytotoxic T lymphocytes (CTLs) and preventing T-cell infiltration in tumors with reduced infiltration of CTLs. The Subclass Mapping (SubMap) approach was applied for evaluating the expression similarity between the groups and the distinct responses to ICIs (Hoshida et al., 2007). Based on the GSEA approach, the degree of commonality between the groups was deduced. Adjusted *p*-value <0.05 indicated significant similarity between the groups.

### Drug Sensitivity Analysis

Using the pRRophetic package (Geeleher et al., 2014), a ridge regression model was built on the basis of the Genomics of Drug Sensitivity in Cancer (GDSC) cell line expression profiles (Yang et al., 2013). The half-maximal inhibitory concentration (IC_50_) values of compounds were estimated across GC specimens.

### Mutational Analysis

Through the maftools package (Mayakonda et al., 2018), somatic variants were analyzed and the overall mutation status was compared between the two phenotypes. Moreover, the top 20 mutated genes were visualized. Through the GISTIC2.0 approach, the recurrently amplified and deleted regions were defined (Mermel et al., 2011).

### Differential Expression Analysis

Through the limma package (Ritchie et al., 2015), the significantly altered genes between the phenotypes were identified according to the following threshold: |log2fold change| > 1 and adjusted *p*-value < 0.05. The *p*-value from Benjamini–Hochberg correction was adjusted for multiple comparisons by a false discovery rate.

### Enrichment Analysis

Gene Ontology (GO) enrichment and Kyoto Encyclopedia of Genes and Genomes (KEGG) pathway analysis were conducted *via* the clusterprofiler package (Yu et al., 2012). With the criteria of adjusted *p*-value < 0.05, significant GO terms and KEGG pathways were screened. The Gene Set Enrichment Analysis (GSEA) was also carried out (Subramanian et al., 2005). The gene set “c2.cp.kegg.v6.2.symbols.gmt” was chosen as the reference.

### Prognostic Signature Construction

The Least Absolute Shrinkage and Selection Operator (LASSO) is a penalized regression analysis that can screen variables from high dimensional data to build risk signatures. Herein, the LASSO analysis was conducted in TCGA cohort to determine the most valuable genes in GC prognosis. The optimal value of the tuning parameter (*λ*) was identified after a ten-fold cross-verification utilizing the minimum and 1- standard error (SE) criteria. The prognostic signature was built by multivariate Cox regression analysis. On the basis of the signature, the risk score was constructed in line with the following formula: risk score = $\underset{i}{\sum }coefficient\;of\;gene\;i\;*expression\;of\;gene\;i$ . GC patients were equally classified into high- and low-risk groups in accordance with the median value of the risk score. The prediction accuracy of the signature was evaluated *via* time-independent receiver operating characteristic (ROC) curves. Moreover, the prognostic value of the signature was externally verified in the GSE15459 cohort.

### Nomogram Establishment

Univariate Cox regression analysis on the clinical features and prognostic signature was conducted in TCGA cohort. The significant prognostic factors with *p*-value < 0.05 were incorporated into the multivariate Cox regression analysis. Through the rms package, the nomogram was built by incorporating factors with prediction significance (*p*-value < 0.05) from the multivariate analysis. Time-dependent ROC curves were drawn for determining the prediction accuracy of the nomogram. A calibration plot was used for assessing the agreement between the predicted and actual outcomes.

### Patients and Specimens

In total, fresh-frozen 20 paired GC and para-carcinoma tissues were acquired with signed informed consent from Xiamen Haicang Hospital. All patients did not receive any treatment before surgery. All procedures involving human specimens gained the approval of the Ethics Committee of Xiamen Haicang Hospital (KY-2020014).

### Quantitative Real-Time Polymerase Chain Reaction

Total RNA extraction from tissues was implemented with a Trizol kit in line with the manufacturer’s instructions. Afterward, the extracted RNA was reverse transcribed into cDNA. qRT-PCR was conducted with a LightCycler 480 system (Roche, Germany). The sequences of primers used for qRT-PCR were as follows: GPX3, 5′-GCC​GGG​GAC​AAG​AGA​AGT-3′ (forward) and 5′-GAG​GAC​GTA​TTT​GCC​AGC​AT-3′ (reverse); RGS2, 5′-AAG​ATT​GGA​AGA​CCC​GTT​TGA​G-3′ (forward) and 5′-GCA​AGA​CCA​TAT​TTG​CTG​GCT-3′ (reverse); MATN3, 5′-TCT​CCC​GGA​TAA​TCG​ACA​CTC-3′ (forward) and 5′-CAA​GGG​TGT​GAT​TCG​ACC​CA-3′ (reverse); SLC7A2, 5′-GAC​CTT​TGC​CCG​ATG​TCT​GAT-3′ (forward) and 5′-AGC​AGC​GGC​ATA​ATT​TGG​TGT-3′ (reverse); SNCG, 5′-TGA​GCA​GCG​TCA​ACA​CTG​TG-3′ (forward) and 5′-GAG​GTG​ACC​GCG​ATG​TTC​TC-3′ (reverse); and GAPDH, 5′-CTG​GGC​TAC​ACT​GAG​CAC​C-3′ (forward) and 5′-AAG​TGG​TCG​TTG​AGG​GCA​ATG-3′ (reverse). The relative mRNA expression was calculated with the 2^−ΔΔCt^ method.

### Statistical Analysis

All data processing was implemented using R 3.6.1 software. The Kaplan–Meier analysis of OS, disease-free survival (DFS), disease-specific survival (DSS), and progression-free survival (PFS) was conducted and compared with log-rank tests. Student’s t-test and Wilcoxon test were performed to conduct the comparisons of the two groups. Pearson and Spearman correlation tests were applied to evaluate the associations between variables. Through the Gene Set Cancer Analysis web-based analysis platform (Liu CJ. et al., 2018), the frequency of the CNV and somatic mutation of genes was analyzed across pan-cancer. Moreover, the Spearman correlation of drug sensitivity and gene expression was analyzed on the basis of the Cancer Therapeutics Response Portal (CTRP) and the GDSC databases. All statistical *p*-values were two-sided, with *p*-value < 0.05 as statistically significant.

## Results

### Construction of Two Exosome-Relevant Phenotypes in Gastric Cancer

**FIGURE 1:**
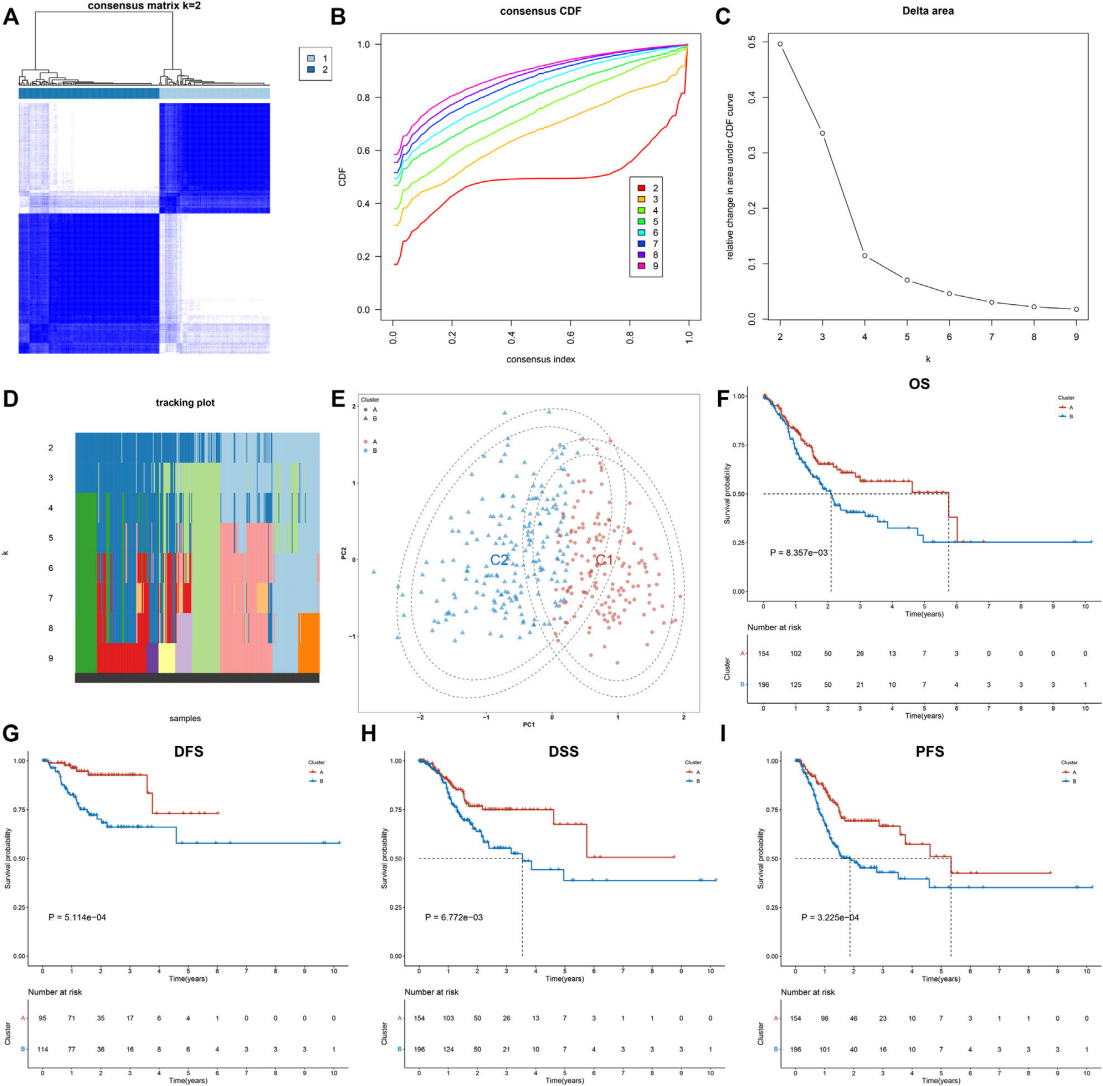
Construction of two exosome-relevant phenotypes with distinct clinical outcomes in GC. **(A)** Consensus score matrix of GC specimens when k = 2. **(B)** CDF of the consensus matrix for each k (indicated by colors). **(C)** Relative alterations in the area under CDF curves. **(D)** Tracking plot for each k. **(E)** PCA plot by expression profiling of 15 prognostic exosome-related genes. Each point indicates each specimen, with unique colors representing exosome-relevant phenotypes. **(F**–**I)** Kaplan–Meier analysis of **(F)** OS, **(G)** DFS, **(H)** DSS, and **(I)** PFS in two exosome-relevant phenotypes.

This study collected 121 exosome-related genes from the published literature. Among them, 15 exosome-related genes were significantly linked to OS outcomes of GC patients (Table 1). On the basis of expression profiling of prognostic exosome-related genes, we conducted a consensus clustering analysis in TCGA cohort, in which GC patients were initially classified into different k (k = 2–9) clusters. In accordance with the consensus matrix, CDF, and tracking plot, the optimal cluster was achieved when k = 2 (Figures 1A–D). The two clusters of GC specimens were separated from one another in accordance with PCA (Figure 1E). Therefore, GC specimens were classified into two exosome-relevant phenotypes, namely, exosome-relevant phenotype A (*n* = 154) and phenotype B (*n* = 196). Exosome-relevant phenotype A presented a remarkable advantage of OS, DFS, DSS, and PFS outcomes relative to phenotype B (Figures 1F–I). To guarantee the reproducibility and robustness of exosome-relevant phenotypes derived from TCGA cohort, this classification was validated in the GSE84437 cohort. The two phenotypes displayed high consistency with TCGA cohort (Supplementary Figures S1A−F).

**TABLE 1:** Prognostic exosome-related genes in GC *via* the univariate analysis.

Exosome-related gene	Hazard ratio	Lower 95% CI	Upper 95% CI	*p*-value
CP	1.345545	1.058641	1.710204	0.015279
CYP11A1	1.473560	1.030760	2.106583	0.033494
RHO	4.395776	1.357214	14.23714	0.013536
ABCB5	2.313005	1.434912	3.728447	0.000577
ADCYAP1	1.511357	1.126606	2.027505	0.005865
MRPL4	0.198812	0.054168	0.729698	0.014893
ADRA1B	1.736174	1.261456	2.389539	0.000711
CD82	0.386935	0.161835	0.925132	0.032766
POSTN	2.154474	1.158856	4.005467	0.015268
HTR7	1.798705	1.116122	2.898735	0.015901
CYP19A1	1.993157	1.347986	2.947118	0.000547
DUSP1	3.428910	1.520732	7.731423	0.002973
ABCC9	1.411726	1.050760	1.896693	0.022103
HRNR	4.134901	1.015019	16.84442	0.047618
DOK7	0.668056	0.501564	0.889813	0.005812

### Distinct Immunological Status in Exosome-Related Phenotypes

**FIGURE 2:**
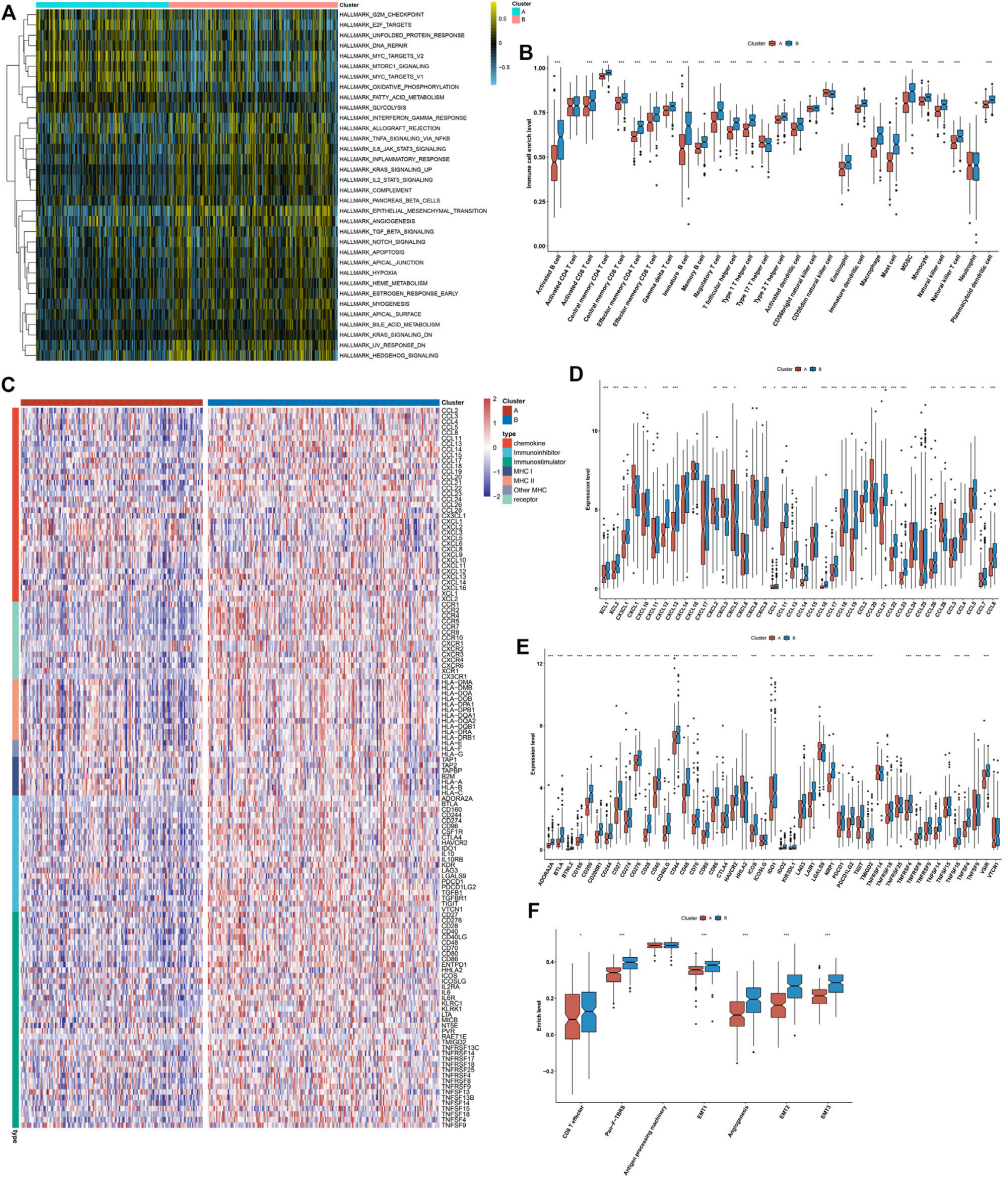
Distinct immunological status in exosome-related phenotypes. **(A)** Visualization of the levels of hallmark pathways in exosome-related phenotypes A and B. **(B)** Differences in the infiltration levels of 28 immune cell types between the phenotypes. **(C)** Visualization of the expression of immunomodulatory molecules (chemokines, immuno-inhibitors, immuno-stimulators, MHC, and receptors) in the two phenotypes. **(D**,**E)** Differences in the expression of **(D)** chemokines and **(E)** immune checkpoints between phenotypes. **(F)** Comparisons of the levels of immune and stromal pathways between phenotypes. ∗*p*-value < 0.05; ∗∗*p*-value < 0.01; and ∗∗∗*p*-value < 0.001.

**FIGURE 3:**
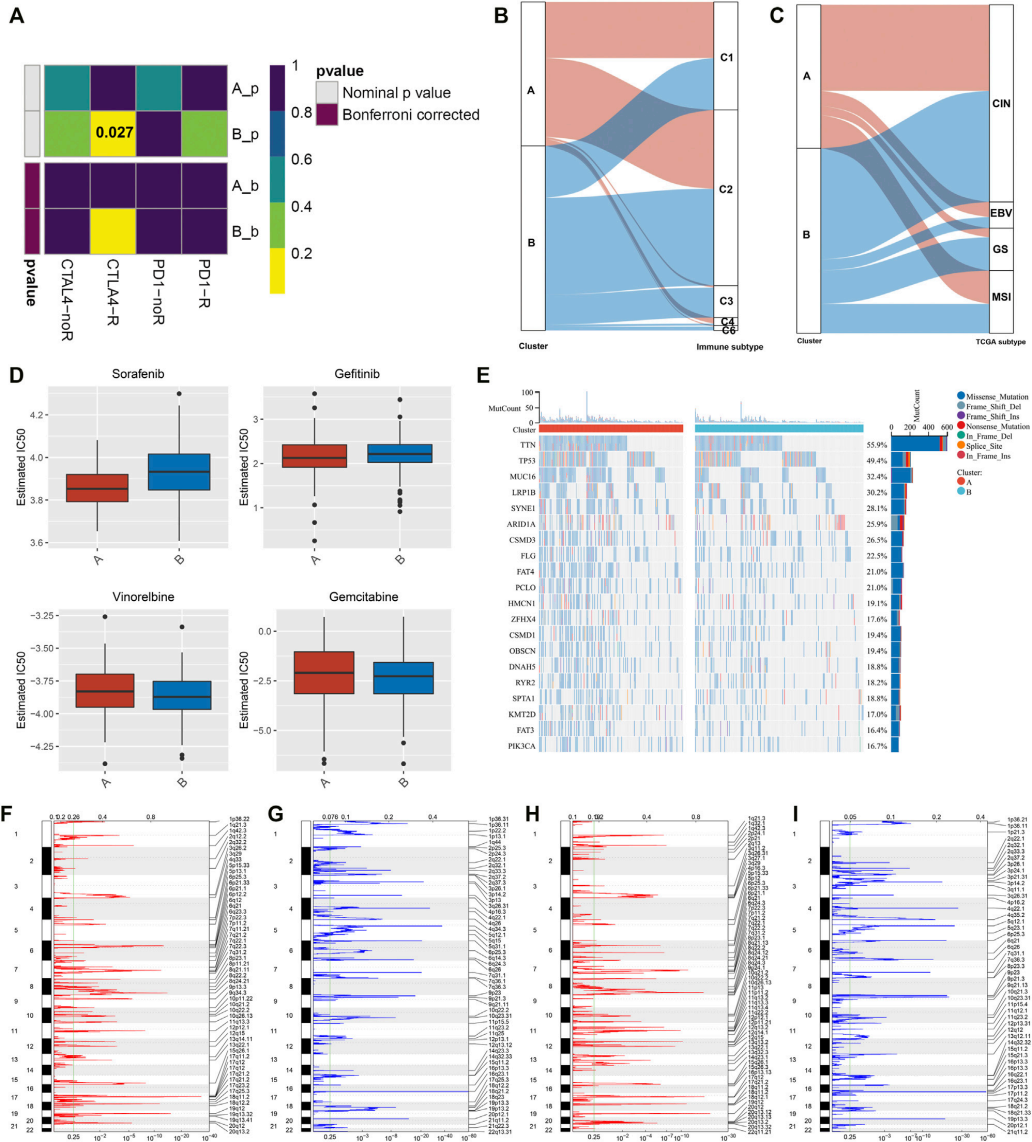
Responses to ICIs, interplay with immunological and molecular subtypes, drug sensitivity, and genomic alterations in exosome-related phenotypes. **(A)** Submap analysis of the similarity of the expression profiling between GC patients in TCGA cohort and 47 previous melanoma subjects who received ICIs. **(B)** Alluvial diagram depicting the interactions between the two exosome-related phenotypes and immunological subtypes. **(C)** Alluvial diagram depicting the interactions between two exosome-related phenotypes and molecular subtypes of GC. **(D)** Differences in estimated IC_50_ values of sorafenib, gefitinib, vinorelbine, and gemcitabine between the exosome-related phenotypes. **(E)** Mutational frequency of the top 20 genes in two exosome-related phenotypes. Gene symbols are ranked in accordance with the frequency of gene mutation. Each column indicates each specimen and the upper panel indicates TMB. **(F**–**I)** Somatic copy number alterations in exosome-related phenotype **(F**,**G)** A and **(H**,**I)** phenotype B. On the x-axis, focal amplification or deletion is separately represented by red or blue bars. The position of chromosomes is shown along the y-axis. The green line indicates the significance threshold of q < 0.25.

This study further investigated the specific biological mechanisms and immunological status of each phenotype in TCGA cohort. The heterogeneity in the activation of hallmark pathways was observed in two exosome-related phenotypes. As shown in Figure 2A, most hallmark pathways were activated in exosome-related phenotype B relative to phenotype A, such as inflammatory or immune activation pathways (interferon-gamma response, allograft rejection, IL6-JAK-STAT3 signaling, inflammatory response, IL2-STAT5 signaling, complement, etc.), stromal activation pathways (EMT, angiogenesis, etc.), and tumorigenic pathways (hedgehog signaling, hypoxia, Notch signaling, TGF-beta signaling, etc.). Moreover, exosome-related phenotype B displayed remarkably higher immune cell infiltration within the TME relative to phenotype A (Figure 2B). In Figures 2C,D, most immunomodulatory molecules (chemokines, immuno-inhibitors, immuno-stimulators, MHC, and receptors) displayed a prominently higher expression in exosome-related phenotype B than phenotype A. We also noted that immune checkpoints were markedly upregulated in exosome-related phenotype B relative to phenotype A (Figure 2E). Our ssGSEA results also confirmed the activation of CD8^+^ T effector, pan-F-TBRS, EMT1-3, and angiogenesis in phenotype B. Overall, exosome-related phenotype B presented an inflamed TME in comparison to phenotype A (Figure 2F). This study applied the submap approach to compare the similarity of the expression profiling between exosome-related phenotypes and 47 melanoma patients who received ICIs. Our results showed that GC patients in exosome-related phenotype B presented higher responses to anti-CTLA4 therapy (Figure 3A).

### Interplay Between Exosome-Related Phenotypes and Immunological and Molecular Subtypes of Gastric Cancer

We observed the interactions between exosome-related phenotypes and immunological subtypes. In Figure 3B, exosome-related phenotypes spanned five of the six immunological subtypes, including C1-wound healing, C2-interferon (IFN)-γ dominant, C3-inflammatory, C4-lymphocyte depleted, and C6-transforming growth factor-β (TGF-β) dominant subtypes. The relatively equal distribution of exosome-related phenotypes was found in C1 and C2 subtypes. Further observation found that C3 and C6 subtypes were particularly dominant in exosome-related phenotype B, while C4 was enriched in exosome-related phenotype A. Thereafter, we evaluated the interactions between exosome-related phenotypes and molecular subtypes. It was found that exosome-related phenotypes spanned chromosomal instability (CIN), Epstein–Barr virus (EBV), genomic stability (GS), and microsatellite instability (MSI) (Figure 3C). However, there was no substantial heterogeneity in the distribution of exosome-related phenotypes. Further investigation showed that the GS subtype was remarkably dominant in exosome-related phenotype B. Overall, exosome-related phenotypes were linked to immunological and molecular subtypes of GC.

### Drug Sensitivity in Exosome-Related Phenotypes

Further analysis was conducted to evaluate the differences in sensitivity to sorafenib, gefitinib, vinorelbine, and gemcitabine between exosome-related phenotypes. As shown in Figure 3D, there were remarkably lower IC_50_ values of sorafenib and gefitinib in exosome-related phenotype A relative to phenotype B, indicating that GC patients in exosome-related phenotype A were more likely to respond to sorafenib and gefitinib. We also noted that exosome-related phenotype B had significantly lower IC_50_ values of vinorelbine and gemcitabine in comparison to exosome-related phenotype A, indicating that patients in exosome-related phenotype B presented higher sensitivity to vinorelbine and gemcitabine.

### Landscape of Genomic Alterations in Exosome-Related Phenotypes

Somatic mutations in the two exosome-related phenotypes were investigated. There were substantial differences in gene mutations between the phenotypes (Figure 3E). TTN (55.9%), TP53 (49.4%), MUC16 (32.4%), and LRP1B (30.2%) were the most frequently mutated genes. Higher TMB was investigated in exosome-related phenotype A relative to phenotype B. Thereafter, an analysis of CNV was presented in two phenotypes. No substantial difference in copy number-amplification was investigated between the phenotypes, but exosome-related phenotype A displayed a higher frequency of copy number-deletion relative to phenotype B (Figures 3F–I).

### Screening Significantly Altered Genes Between Exosome-Related Phenotypes

**FIGURE 4:**
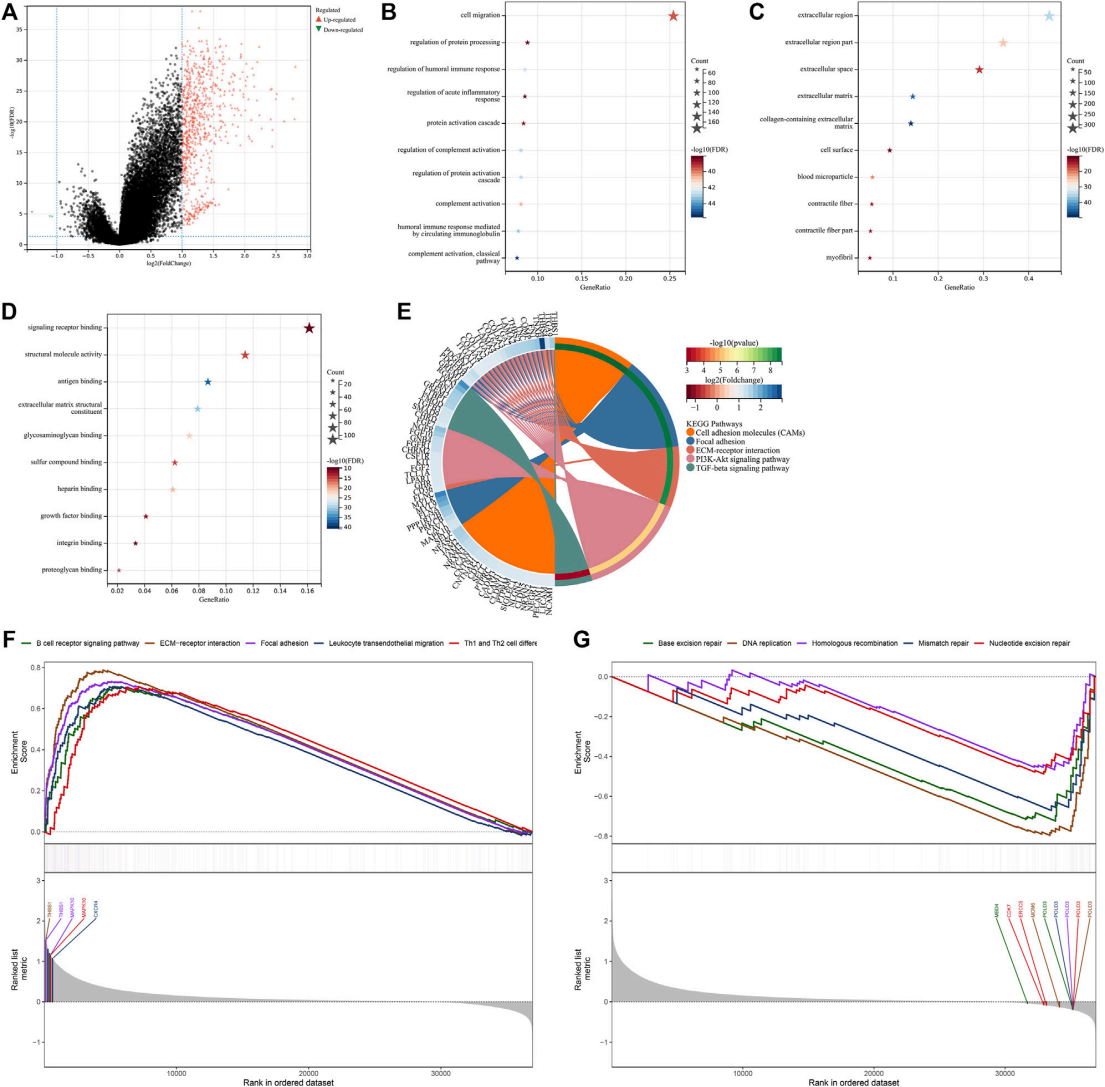
Screening significantly altered genes between the exosome-related phenotypes and analysis of their biological implications. **(A)** Volcano plot for up- and downregulated genes in exosome-related phenotype A when compared with phenotype B. **(B**–**D)** Visualization of the top ten **(B)** biological processes, **(C)** cellular components, and **(D)** molecular functions, respectively. **(E)** Visualization of the top five KEGG pathways enriched by significantly altered genes. **(F)** GSEA for the signaling pathways positively correlated to significantly altered genes. **(G)** GSEA for the signaling pathways negatively associated with significantly altered genes.

For finding the genes most correlated to exosome-related phenotypes, we conducted differential expression analysis between two phenotypes. Under the threshold of |log2fold change| > 1 and adjusted *p*-value < 0.05, 773 significantly altered genes were determined (Figure 4A; Supplementary Table S2). Further observation showed that these significantly altered genes were linked to cell migration, immune or inflammatory response, protein activation (Figure 4B), and extracellular components (Figure 4C). Moreover, the significantly altered genes were correlated to molecular functions of signaling receptor binding, structural molecule activity, antigen binding, extracellular matrix structural constituent, etc. (Figure 4D). It was also found that the significantly altered genes were enriched in tumorigenic pathways such as cell adhesion molecules, focal adhesion, ECM-receptor interaction, and PI3K-Akt and TGF-beta signaling pathways (Figure 4E). For validating the reliability of the KEGG pathway analysis, we conducted the GSEA based on the significantly altered genes. As shown in Figure 4F, the significantly altered genes displayed positive interactions with the B-cell receptor signaling pathway, ECM-receptor interaction, focal adhesion, leukocyte trans-endothelial migration, and Th1 and Th2 cell differentiation, whereas they were negatively linked to base excision repair, DNA replication, homologous recombination, mismatch repair, and nucleotide excision repair (Figure 4G). Thus, the aforementioned significantly altered genes might exert important roles in GC.

### Construction of the Exosome-Based Gene Signature

**FIGURE 5:**
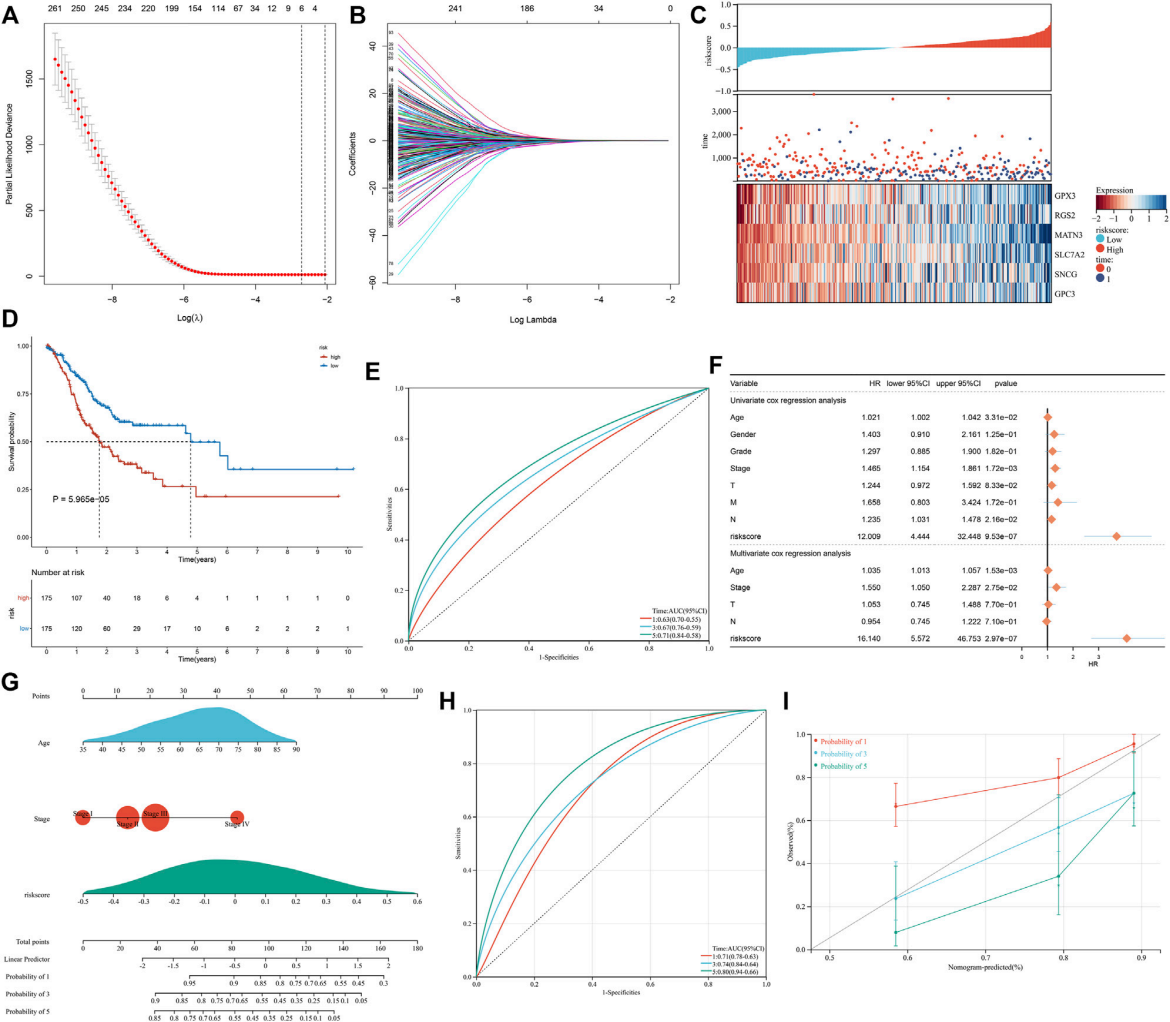
Construction of the exosome-based gene signature for GC in TCGA cohort. **(A)** Partial likelihood deviance in the LASSO regression model *via* the ten-fold cross verification. The vertical dotted lines represent the optimal values utilizing the minimum and 1-SE criteria. **(B)** LASSO coefficient profiling. **(C)** Overview of the risk score distribution (upper), survival status (middle), and expression of genes that made up the signature (lower). **(D)** Kaplan–Meier curves of OS in TCGA cohort, divided into high- and low-risk groups. **(E)** Time-independent ROC curves showing the prediction accuracy of this signature. **(F)** Forest plot showing the associations of risk score and clinical features with OS according to uni- and multivariate Cox regression models. **(G)** Construction of the nomogram that incorporated the risk score, age, and histological staging. **(H)** Time-independent ROC curves showing the prediction accuracy of the nomogram. **(I)** Calibration plot showing the agreement between the nomogram-predicted and actual 1-, 3-, and 5-year outcomes. The x-axis represents the nomogram-predicted OS, and the y-axis represents the actual OS. The ideal performance of the nomogram is shown by the dashed line along the 45°line.

The univariate analysis showed that 266 significantly altered genes were significantly linked with GC prognosis (Supplementary Table S3). The LASSO analysis was conducted based on the prognostic significantly altered genes. Through the minimum and 1-SE criteria, five genes (GPX3, RGS2, MATN3, SLC7A2, and SNCG) were chosen to establish the exosome-based gene signature in TCGA cohort (Figures 5A,B). The risk score of each patient was calculated, and all patients were stratified into high- and low-risk groups following the median value (Figure 5C). The high-risk group had more cases with dead status and reduced expression of the aforementioned five genes. For evaluating the prognostic implication of this model, the difference in the OS between the groups was estimated. As shown in Figure 5D, high-risk patients presented a significantly reduced OS than their counterparts in TCGA cohort. Time-independent ROC curves demonstrated that the signature was accurately predictive of GC patients’ OS (Figure 5E). Moreover, the associations between the signature and OS were evaluated *via* uni- and multivariate analysis. As shown in Figure 5F, the signature, age, and stage were independent risk factors of OS. For examining the robustness of the signature, the prediction performance was tested in the GSE15459 cohort. With the same formula, GC patients were stratified into high- and low-risk groups (Supplementary Figure S2A). Consistent with the outcomes of TCGA cohort, high-risk patients presented a significantly poorer OS than their counterparts (Supplementary Figure S2B), with a high prediction accuracy (Supplementary Figure S2C). For providing clinicians with a quantitative approach for predicting GC patients’ outcomes, the nomogram was built by incorporating the aforementioned independent risk factors (Figure 5G). On the basis of the nomogram, a score was calculated for an individual patient for predicting the 1-, 3-, and 5-year OS. Further observation showed that the exosome-based gene signature contributed to the most risk points in comparison to age and histological staging. Time-independent ROC curves demonstrated that the nomogram presented high accuracy in predicting OS (Figure 5H). The calibration plot showed that the nomogram-predicted OS fit well with the actual outcomes (Figure 5I). Thus, the exosome-based gene signature could optimize risk stratification and accurately predict GC patients’ OS.

### Exosome-Based Gene Signature is Linked to Stromal Activation and Immunosuppression

**FIGURE 6:**
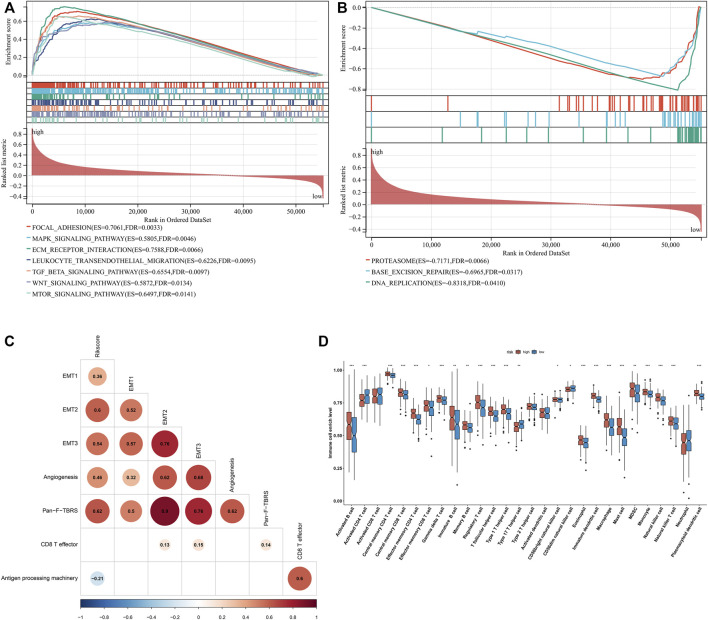
Exosome-based gene signature is linked to stromal activation and immunosuppression in GC. **(A)** GSEA showing the signaling pathways positively linked to the exosome-based gene signature. **(B)** GSEA showing the signaling pathways negatively associated with the signature. **(C)** Associations of the signature with stromal and immune activation pathways that were quantified by the ssGSEA approach. **(D)** Comparisons of the relative abundance levels of 28 immune cell types in the high-risk group relative to the low-risk group. ∗*p*-value < 0.05; ∗∗*p*-value < 0.01; and ∗∗∗*p*-value < 0.001.

Further investigation showed that the exosome-based gene signature presented positive interactions with stromal activation pathways, such as focal adhesions, ECM receptor interaction, TGF-beta, and WNT and mTOR signaling pathways (Figure 6A). Moreover, this signature was negatively linked to proteasome, base excision repair, and DNA replication (Figure 6B). As shown in Figure 6C, the exosome-based gene signature was positively correlated to EMT1-3, angiogenesis, and pan-F-TBRS, consistent with the GSEA results. It was also found that this signature displayed a negative association with antigen processing machinery. Immunosuppressive myeloid cells such as MDSC, tumor-associated macrophages, and regulatory T cells displayed significantly higher infiltration in the high-risk group relative to the low-risk group (Figure 6D). Overall, the exosome-based gene signature was linked to stromal activation and immunosuppression in GC.

### Landscape of Genetic alterations, Drug Sensitivity, and Immune Cell Infiltration in the Exosome-Based Gene Signature

**FIGURE 7:**
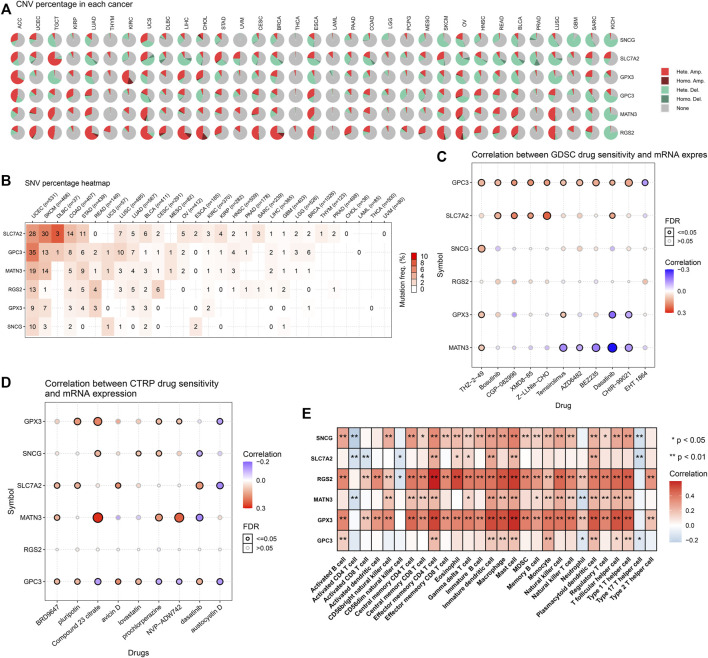
Landscape of genetic alterations, drug sensitivity, and immune cell infiltration in the exosome-based gene signature. **(A)** Percentage of CNVs of each gene in the exosome-based gene signature across pan-cancer. **(B)** Mutation frequencies of each gene in the signature across pan-cancer. **(C)** Associations of mRNA expression of each gene with sensitivity to small molecular compounds in accordance with the GDSC database. **(D)** Associations of mRNA expression of each gene with sensitivity to small molecular compounds in accordance with the CTRP database. **(E)** Associations of mRNA expression of each gene with immune cell infiltration in the TME.

There were widespread amplifications and deletions of five genes (GPX3, RGS2, MATN3, SLC7A2, and SNCG) across pan-cancer (Figure 7A). Most cancer types had a relatively high mutation frequency of the aforementioned genes (Figure 7B). On the basis of the GDSC and CTRP projects, all of them were remarkably linked to the sensitivity to THZ-2-49, Bosutinib, CGP-082996, XMD8-85, Z-LLNle-CHO, Temsirolimus, AZD6482, BEZ235, Dasatinib, CHIR-99021, EHT 1864 (Figure 7C), BRD9647, pluripotin, compound 23 citrate, avicin D, lovastatin, prochlorperazine, NVP-ADW742, dasatinib, and austocystin D (Figure 7D). Moreover, they presented prominent correlations to the infiltrations of immune cells (activated B cell, activated CD4 T cell, activated CD8 T cell, activated dendritic cell, CD56bright natural killer cell, CD56dim natural killer cell, central memory CD4 T cell, central memory CD8 T cell, effector memory CD4 T cell, effector memory CD8 T cell, eosinophil, gamma delta T cell, immature B cell, macrophage, mast cell, MDSC, memory B cell, monocyte, natural killer cell, natural killer T cell, neutrophil, plasmacytoid dendritic cell, regulatory T cell, T follicular helper cell, type 1 helper cell, type 17 helper cell, and type 2 helper cell) within the TME of GC (Figure 7E). The aforementioned data indicated the implications of GPX3, RGS2, MATN3, SLC7A2, and SNCG in GC.

### Experimental Verification of Altered Genes From Exosome-Based Phenotypes

**FIGURE 8:**
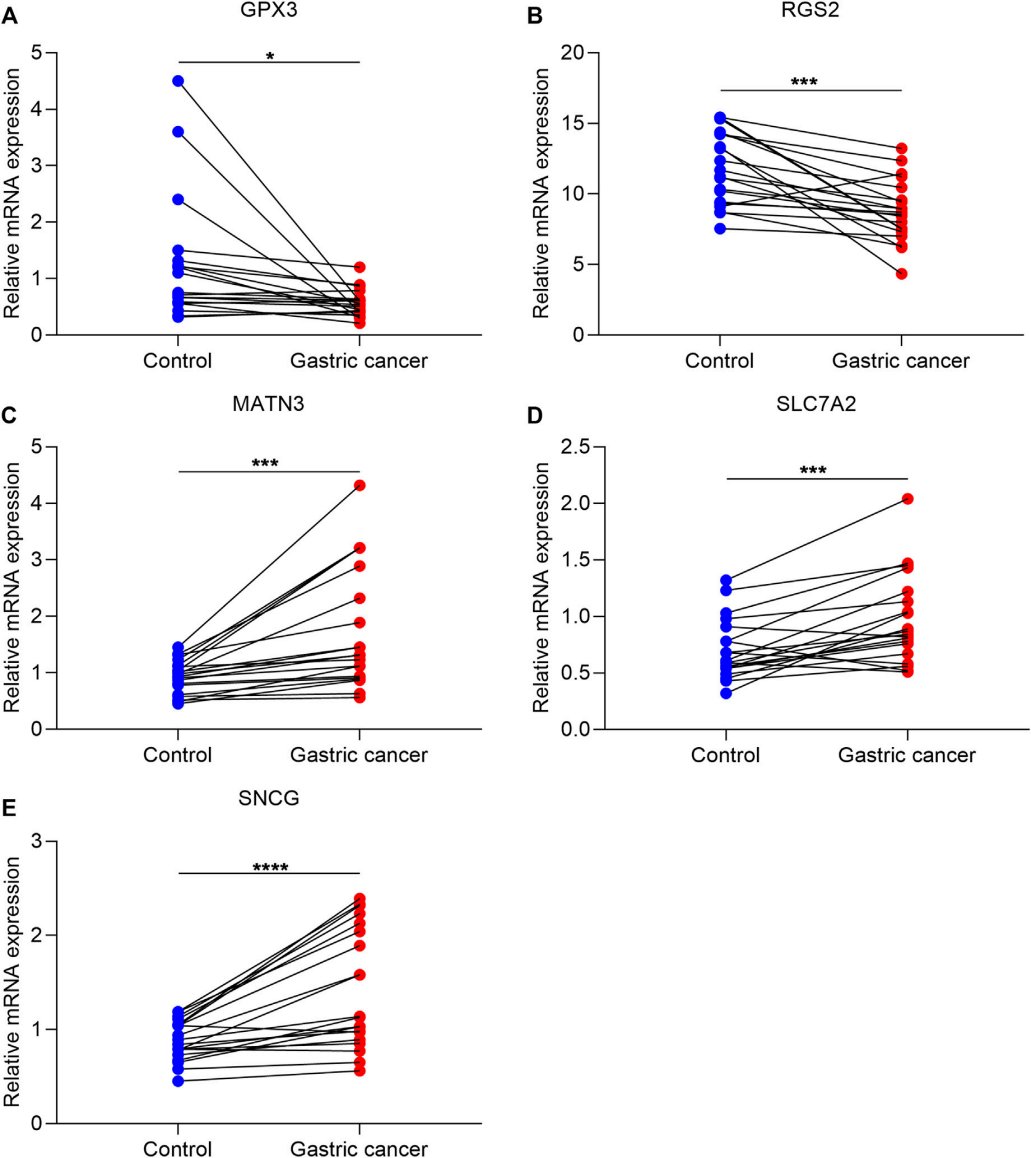
Experimental verification of altered genes from exosome-based phenotypes. **(A**–**E)** qRT-PCR for validating the mRNA expression of GPX3, RGS2, MATN3, SLC7A2, and SNCG in 20 paired GC and para-carcinoma tissues. ∗*p*-value<0.05; ∗∗∗*p*-value<0.001; and ∗∗∗∗*p*-value<0.0001.

To validate altered genes from the exosome-based phenotypes, we collected 20 paired GC and para-carcinoma tissues. Our qRT-PCR results confirmed that GPX3 and RGS2 were significantly downregulated in GC than in para-carcinoma tissues (Figures 8A,B). In addition, MATN3, SLC7A2, and SNCG were significantly upregulated in GC compared with para-carcinoma tissues (Figures 8C–E).

## Discussion

GC is a highly heterogeneous malignant carcinoma and the classification of GC based on molecular subtypes is essential to personalized therapy (Lin et al., 2020). A few subtype systems have been conducted, such as ACRG and TCGA subtypes (Serra et al., 2019). Nevertheless, the exosome-based classification of GC is not well defined. Herein, we conducted the exosome-based classification on the basis of the expression profiling of 15 prognostic exosome-related genes. The reproducibility of this classification was confirmed in the independent cohort. The exosome-relevant phenotypes we proposed were independent of existing classifications (immune subtypes and TCGA subtypes), indicating that this classification deserves in-depth analysis.

Exosome-related phenotype B had poorer OS, DSS, DFS, and PFS relative to phenotype A. Further analysis uncovered that phenotype B displayed the activation of tumorigenic pathways (hedgehog signaling, hypoxia, Notch signaling, TGF-beta signaling, etc.), contributing to an undesirable prognosis. Immunotherapy presents durable antitumor activity against GC therapy. Nevertheless, this therapy still faces many challenges (Shitara et al., 2018; Shitara et al., 2020; Janjigian et al., 2021). It has been realized that TME is of complexity and diversity concerning immunological status (Liu et al., 2020). Thus, the prediction of the responses to ICIs on the basis of the TME cell infiltrations represents an important procedure to enhance the efficacy of current ICIs and to exploit new immunotherapeutic regimens (Zhang et al., 2020). The immune evasion mechanisms exert crucial roles in immunotherapy (Kong et al., 2020). Exosome-related phenotype B displayed the activation of inflammatory or immune activation pathways (interferon-gamma response, allograft rejection, IL6-JAK-STAT3 signaling, inflammatory response, IL2-STAT5 signaling, complement, CD8^+^ T effector, etc.) and stromal activation pathways (EMT, angiogenesis, etc.). Moreover, exosome-related phenotype B displayed remarkably higher immune cell infiltration within the TME and higher expression of immunomodulatory molecules (chemokines, immuno-inhibitors, immuno-stimulators, MHC, and receptors) and immune checkpoints relative to phenotype A. Thus, exosome-related phenotype B had an inflamed TME. A clinical trial showed the low responses of GC patients to tremelimumab, an anti-CTLA4 inhibitor (Kelly et al., 2020). It was predicted that GC patients with exosome-related phenotype B displayed higher responses to anti-CTLA4 therapy. This also demonstrated that the exosome-related phenotype might be an underlying indicator for predicting the response to ICIs.

Higher somatic mutation and copy number-deletion occurred in exosome-related phenotype B. Despite ICIs being a key discovery in GC treatment, chemotherapy remains an important regimen for postoperative treatment (Qiu et al., 2020). Exosome-related phenotype A had higher sensitivity to sorafenib and gefitinib, while exosome-related phenotype B had higher sensitivity to vinorelbine and gemcitabine. Experimental evidence has demonstrated that exosomes derived from tumor cells can mediate the resistance to sorafenib (Qu et al., 2016), gefitinib (Kang et al., 2018), and gemcitabine (Mikamori et al., 2017). Thus, this classification might predict sensitivity to sorafenib, gefitinib, vinorelbine, and gemcitabine.

We established the exosome-based gene signature (comprising GPX3, RGS2, MATN3, SLC7A2, and SNCG) that was an independent prognostic indicator of GC. Moreover, the nomogram was built by incorporating this exosome-based gene signature and age and histological staging, which provided clinicians with a quantitative approach for predicting GC patients’ outcomes. The signature was linked to stromal activation and immunosuppression of GC. A few limitations should be pointed out in our study. First, our analysis was only focused on exosome-related genes in GC tissues. Second, the possibility of selection bias in this retrospective study cannot be ruled out. Third, GC is a highly heterogeneous malignancy. Two exosome-based phenotypes to predict the responses to ICIs might be inadequate.

## Conclusion

Collectively, we constructed two exosome-relevant phenotypes in GC based on exosome-related genes, characterized by distinct survival outcomes, immunological status, and drug sensitivity. In addition, we determined and experimentally verified five altered genes (GPX3, RGS2, MATN3, SLC7A2, and SNCG) from exosome-based phenotypes. Based on the aforementioned genes, we established the exosome-based gene signature that could accurately predict patients’ prognosis and was linked to stromal activation and immunosuppression. Altogether, our findings demonstrated the molecular mechanisms underlying exosomes in GC, which could assist us in comprehending the immune infiltration and immune evasion mechanisms in GC. The exosome-based phenotype could be used for stratifying GC patients and identifying patients who might respond to ICIs or chemotherapy.

## Data Availability

The datasets presented in this study can be found in online repositories. The names of the repository/repositories and accession number(s) can be found in the article/Supplementary Material.

## Supplementary Material

Supplementary Figure S1 External validation of two exosome-relevant phenotypes in the GSE84437 cohort. **(A)** Consensus score matrix of GC specimens when k = 2. **(B)** CDF of consensus matrix for each k (indicated by colors). **(C)** Relative alterations in the area under CDF curves. **(D)** Tracking plot for each k. **(E)** PCA plot by expression profiling of 15 prognostic exosome-related genes. Each point indicates each specimen, with unique colors representing exosome-relevant phenotypes. **(F)** Kaplan–Meier analysis of OS in two exosome-relevant phenotypes.

Supplementary Figure S2 External verification of the prognostic implication of the exosome-based gene signature for GC in the GSE15459 cohort. **(A)** Overview of risk score distribution (upper), survival status (middle), and expression of genes that made up the signature (lower). **(B)** Kaplan–Meier curves of OS in the GSE15459 cohort, separated into high- and low-risk groups. **(C)** Time-independent ROC curves showing the prediction accuracy of the model.

Supplementary Table S1 List of exosome-related genes.

Supplementary Table S2 List of significantly altered genes between exosome-related phenotypes.

Supplementary Table S3 List of significantly altered genes that were significantly linked with GC prognosis.

## Abbreviations

GC, gastric cancer; OS, overall survival; ICIs, immune checkpoint inhibitors; TME, tumor microenvironment; ECM, extracellular matrix; CNV, copy number variation; CDF, cumulative distribution function; PCA, principal component analysis; GSVA, Gene Set Variation Analysis; MSigDB, Molecular Signatures Database; ssGSEA, single-sample Gene Set Enrichment Analysis; Pan-F-TBRS, pan-fibroblast TGFb response signature; TIDE, Tumor Immune Dysfunction and Exclusion; CTLs, cytotoxic T lymphocytes; SubMap, Subclass Mapping; GDSC, Genomics of Drug Sensitivity in Cancer; IC_50_, half-maximal inhibitory concentration; GO, Gene Ontology; KEGG, Kyoto Encyclopedia of Genes and Genomes; GSEA, Gene Set Enrichment Analysis; LASSO, Least Absolute Shrinkage and Selection Operator; ROC, receiver operating characteristic; DFS, disease-free survival; DSS, disease-specific survival; PFS, progression-free survival; CTRP, Cancer Therapeutics Response Portal; qRT-PCR, quantitative real-time polymerase chain reaction.
