# Supplementary figures and images for: A Novel Exosome-Relevant Molecular Classification Uncovers Distinct Immune Escape Mechanisms and Genomic Alterations in Gastric Cancer
Source: Front Pharmacol. 2022 Jun 3;13:884090. doi: 10.3389/fphar.2022.884090 (PMC9204030; doi:10.3389/fphar.2022.884090)

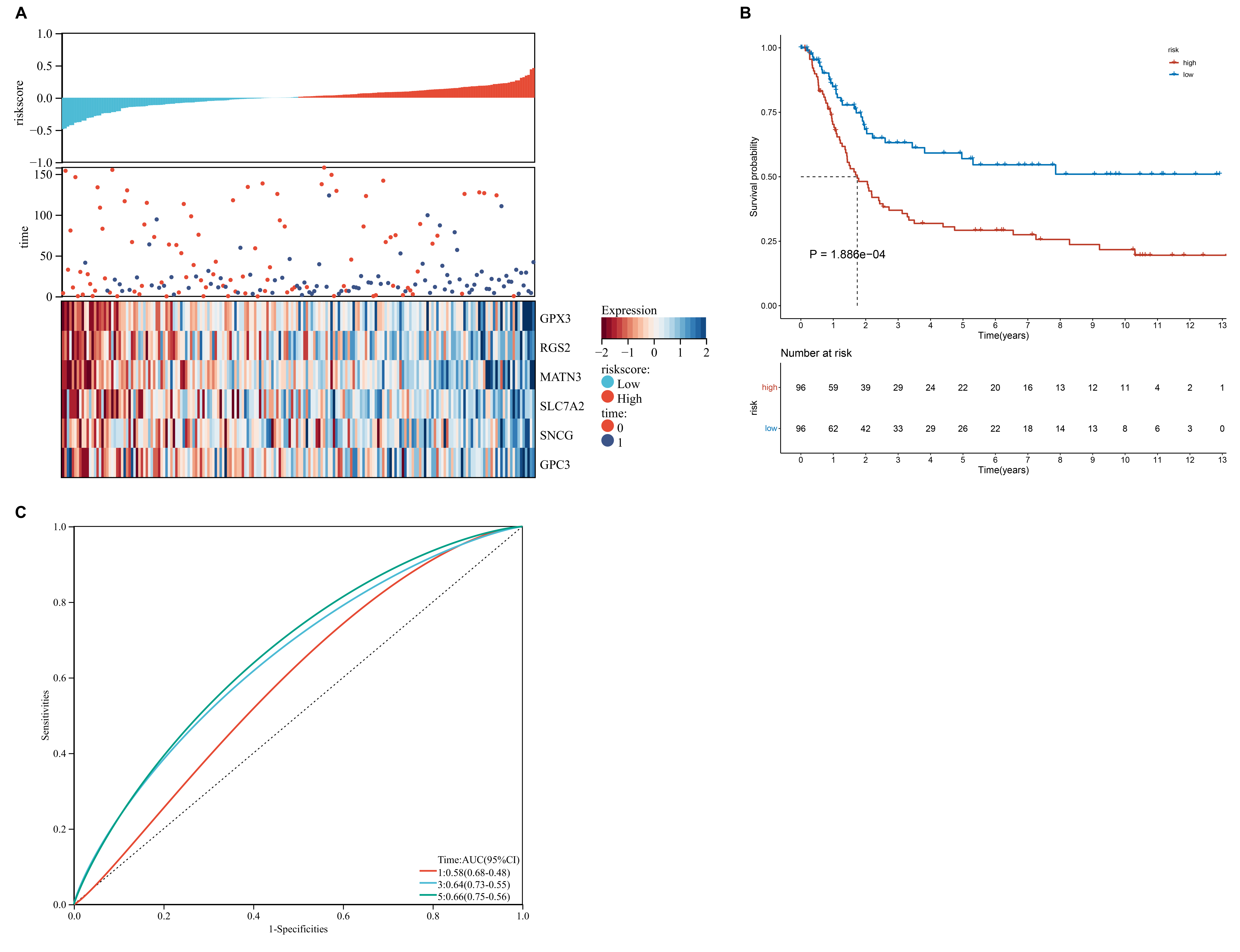

Supplement: Supplementary file 3 [file Image2.TIF]

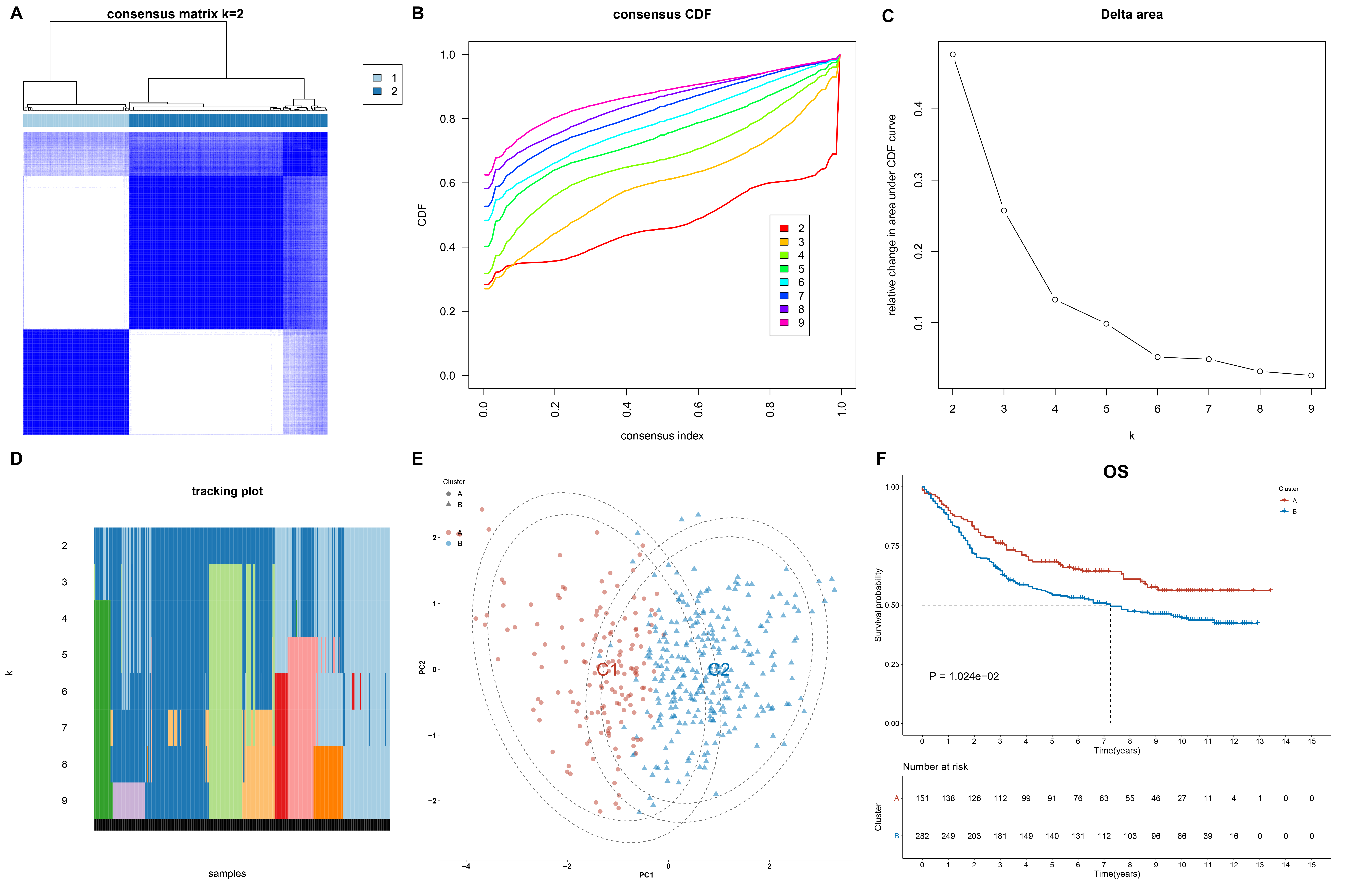

Supplement: Supplementary file 4 [file Image1.TIF]
